# Species-Specific Inhibition of RIG-I Ubiquitination and IFN Induction by the Influenza A Virus NS1 Protein

**DOI:** 10.1371/journal.ppat.1003059

**Published:** 2012-11-29

**Authors:** Ricardo Rajsbaum, Randy A. Albrecht, May K. Wang, Natalya P. Maharaj, Gijs A. Versteeg, Estanislao Nistal-Villán, Adolfo García-Sastre, Michaela U. Gack

**Affiliations:** 1 Department of Microbiology, Mount Sinai School of Medicine, New York, New York, United States of America; 2 Global Health and Emerging Pathogens Institute, Mount Sinai School of Medicine, New York, New York, United States of America; 3 Department of Microbiology and Immunobiology, New England Primate Research Center, Harvard Medical School, Southborough, Massachusetts, United States of America; 4 Department of Medicine, Mount Sinai School of Medicine, New York, New York, United States of America; Johns Hopkins University - Bloomberg School of Public Health, United States of America

## Abstract

Influenza A viruses can adapt to new host species, leading to the emergence of novel pathogenic strains. There is evidence that highly pathogenic viruses encode for non-structural 1 (NS1) proteins that are more efficient in suppressing the host immune response. The NS1 protein inhibits type-I interferon (IFN) production partly by blocking the TRIM25 ubiquitin E3 ligase-mediated Lys63-linked ubiquitination of the viral RNA sensor RIG-I, required for its optimal downstream signaling. In order to understand possible mechanisms of viral adaptation and host tropism, we examined the ability of NS1 encoded by human (Cal04), avian (HK156), swine (SwTx98) and mouse-adapted (PR8) influenza viruses to interact with TRIM25 orthologues from mammalian and avian species. Using co-immunoprecipitation assays we show that human TRIM25 binds to all tested NS1 proteins, whereas the chicken TRIM25 ortholog binds preferentially to the NS1 from the avian virus. Strikingly, none of the NS1 proteins were able to bind mouse TRIM25. Since NS1 can inhibit IFN production in mouse, we tested the impact of TRIM25 and NS1 on RIG-I ubiquitination in mouse cells. While NS1 efficiently suppressed human TRIM25-dependent ubiquitination of RIG-I 2CARD, NS1 inhibited the ubiquitination of full-length mouse RIG-I in a mouse TRIM25-independent manner. Therefore, we tested if the ubiquitin E3 ligase Riplet, which has also been shown to ubiquitinate RIG-I, interacts with NS1. We found that NS1 binds mouse Riplet and inhibits its activity to induce IFN-β in murine cells. Furthermore, NS1 proteins of human but not swine or avian viruses were able to interact with human Riplet, thereby suppressing RIG-I ubiquitination. In conclusion, our results indicate that influenza NS1 protein targets TRIM25 and Riplet ubiquitin E3 ligases in a species-specific manner for the inhibition of RIG-I ubiquitination and antiviral IFN production.

## Author Summary

Influenza viruses cause annual epidemics and occasionally, major global pandemics. To establish productive infection these viruses have mechanisms to evade host immune responses, including the type-I interferon (IFN) response. An important component of the IFN system is the helicase RIG-I that recognizes viral RNA, and is subsequently ubiquitinated by TRIM25 ubiquitin E3 ligase to induce downstream signaling resulting in IFN-α/β production. The NS1 protein of influenza A viruses binds to human TRIM25 and inhibits TRIM25-dependent RIG-I ubiquitination and downstream RIG-I signaling. An important unresolved question is how viruses can inhibit the RIG-I pathway when infecting new hosts. Here we show that while human TRIM25 is able to bind to different NS1 proteins, chicken TRIM25 binds preferentially to the NS1 from an avian virus. Strikingly, mouse TRIM25 was unable to bind NS1. We found that NS1 blocks RIG-I signaling in mouse and human cells by different mechanisms. While NS1 inhibits human TRIM25-mediated RIG-I ubiquitination, in mouse cells NS1 suppresses RIG-I signaling by binding to and inhibiting the ubiquitin E3 ligase Riplet. These results help understand the immune evasion strategies used by influenza virus in different species, and may partly explain the ability of this virus to adapt to different host species.

## Introduction

Influenza A viruses (IAVs) are highly infectious pathogens that have caused major pandemics and annual epidemics with serious economic and health consequences [1], [2]. IAVs are naturally maintained in avian species but they also circulate in humans, horses, dogs and pigs [3]. Although multigenic host range restrictions exist, a combination of viral determinants can ultimately allow a virus to establish infection in a specific host [4]. This is particularly important because, although the current highly pathogenic avian IAVs that have been transmitted to humans lack the ability to spread from human to human, there is current concern that these avian viruses may adapt and develop the ability to spread efficiently among humans. In this respect, recent studies have demonstrated that only a few mutations in the hemagglutinin (HA) allow for transmissibility of highly pathogenic H5N1 viruses in ferrets [5], [6]. Moreover, pigs can be infected with human and avian viruses and provide an environment for reassortment and the generation of new influenza virus strains capable of human transmission [7]. Therefore, it is essential to better understand the mechanisms that allow influenza viruses to adapt to a new host species, in order to predict and protect from future cross-species transmission.

IAV is an enveloped virus that harbors a negative-strand RNA genome encoding eleven different proteins from 8 separate segments [8]. Individual viral proteins play critical roles in species-specific pathogenicity. For example, the hemagglutinin (HA) protein which binds in a species-dependent manner to sialic acid on host cell membranes; the neuraminidase (NA) protein which is important for viral release; and the polymerase components (PA, PB1, PB2) which are important for efficient replication [9]. The non-structural protein 1 (NS1), which is the product of the smallest RNA segment, acts as a virulence factor by inhibiting host immune responses [10]–[15]. An important host innate immune mechanism is the production of type-I interferons (IFNs), which can establish an antiviral state by up-regulating interferon stimulated genes (ISGs) that interfere with distinct steps in the viral life cycle [16], [17].

Type-I IFNs and other pro-inflammatory cytokines are produced when host pattern recognition receptors (PRRs), including Toll-like receptors (TLRs) and RIG-I-like receptors (RLRs), recognize pathogen-associated molecular patterns (PAMPs) such as nucleic acids or other structural components of the microbe [18]–[21]. Viral single-stranded or short double-stranded RNA bearing a 5′-triphosphate is recognized by the cytosolic RNA helicase RIG-I [22], [23]. In addition, next-generation RNA sequencing revealed that in influenza A virus infected cells RIG-I preferentially associated with the shorter genomic segments as well as subgenomic defective interfering particles [24]. RIG-I is composed of two N-terminal caspase recruitment domains (CARDs), a central DExD/H box helicase/ATPase, and a C-terminal regulatory domain (CTD/RD). Under normal conditions, RIG-I is in an inactive, closed conformation. Upon RNA recognition, exposure of the N-terminal CARDs of human RIG-I leads to Lys63-linked ubiquitination on Lys172 induced by tripartite motif 25 (TRIM25) ubiquitin E3 ligase. The ubiquitinated CARDs then allow RIG-I binding to MAVS (also called IPS-1, Cardif, or VISA) and thereby downstream signaling for IRF3 phosphorylation and type-I IFN production [25]. Another ubiquitin E3 ligase, Riplet (or RNF135), has also been reported to ubiquitinate RIG-I and promote its signal transducing ability for antiviral IFN production [26], [27]. Specifically, Riplet was shown to activate RIG-I by inducing Lys63-linked ubiquitination of the lysine residues 849 and 851 in the RIG-I CTD [26], [27].

Many viruses are equipped with specialized mechanisms to suppress the host type-I IFN system. Influenza A viruses through their NS1 protein can inhibit type-I IFN production by various mechanisms, including inhibition of RIG-I [23], [28]–[30] and, in some cases suppressing general host gene expression by targeting the 30 kDa subunit of cleavage and polyadenylation specificity factor (CPSF30) [31]–[33]. The importance of this mechanism of antagonism is further supported by the fact that some highly pathogenic viruses encode NS1 proteins that are more efficient in suppressing the host antiviral response. For example, the NS1 protein of the highly pathogenic 1918 virus blocked the expression of IFN-regulated genes more efficiently than the NS1 from influenza A/WSN/33 virus [11]. In addition, the NS1 gene of a human IAV, when passaged in mouse, accumulated adaptive mutations that resulted in increased virulence and IFN antagonism [34]. Importantly, in addition to NS1, also other viral genes, including PA, PB1, PB2 and PB1-F2 have been shown to exert IFN-antagonistic functions [35]–[37].

We have recently uncovered the mechanism of RIG-I inhibition by NS1 [38]. NS1 binds to the central coiled-coil (CCD) region of TRIM25 ubiquitin E3 ligase, blocking its oligomerization and enzymatic activity to induce Lys63-linked ubiquitination of the RIG-I CARDs. NS1 thereby inhibits RIG-I CARD-dependent downstream signaling [38]. However there are differences in PRR signaling components of different species that could impact NS1 immunosuppressive function. For example, mouse RIG-I lacks Lys172, which has been previously shown to be essential for ubiquitination-dependent activation of human RIG-I [25]. In addition, it appears that chicken lacks a functional RIG-I gene [39]. This host specificity in the RIG-I antiviral signaling pathway may provide a barrier for new incoming viruses, which in turn will need to adapt to inhibit these host-specific immune responses.

In this study, we examined the ability of NS1 encoded by human (Cal04), avian (HK156), swine (SwTx98), and mouse (PR8) adapted influenza A viruses to interact with TRIM25 orthologues from mammalian and avian species, and the impact that this interaction has in regulating the RIG-I-mediated type-I IFN response. We found that while NS1 efficiently inhibits TRIM25-mediated RIG-I ubiquitination and activation in human cells, in murine cells NS1 targets the ubiquitin E3 ligase Riplet to suppress RIG-I ubiquitination and signal transducing activity. Furthermore, we identified that the NS1 proteins of human viruses have evolved to bind and antagonize both human TRIM25 and human Riplet for the potent inhibition of the RIG-I-mediated IFN response.

## Results

### Influenza A Virus NS1 Protein Interacts with TRIM25 in a Species-specific Manner

Given that mammalian and avian TRIM25 proteins show significant differences in their CCD (Supplemental Figure S1A), that in the case of human TRIM25 is necessary and sufficient for NS1 binding [38], we tested the ability of NS1 encoded by human (A/California/04/09 [Cal04]), avian (A/Hong Kong/156/1997 [HK156]), swine (A/Swine/Texas/4199-2/98 [SwTx98]) and mouse-adapted (A/Puerto Rico/8/34 [PR8]) influenza A viruses to interact with TRIM25 orthologues. To facilitate these studies, we used NS1 proteins that lack the ability to bind CPSF30 and therefore are unable to block general host gene expression [40]. Furthermore, these NS1 proteins have conserved E96/E97 amino acids previously shown to be important for interaction with human TRIM25 (Supplemental Figure S1B) [38].

**Figure 1:**
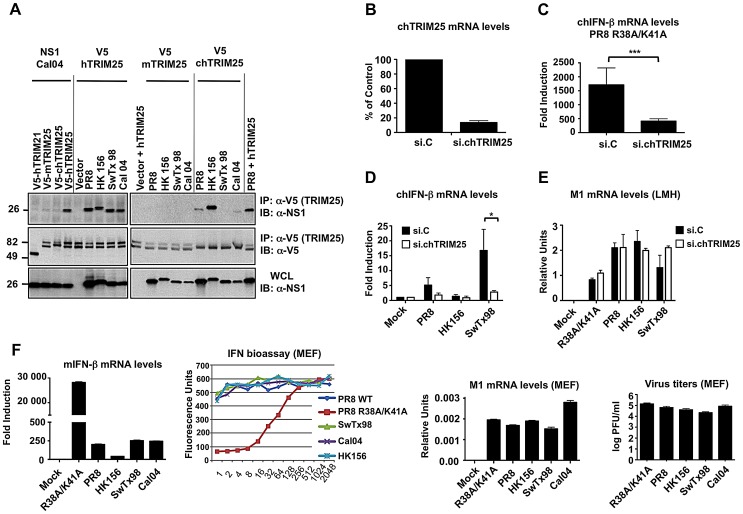
Influenza A Virus NS1 interacts with TRIM25 in a species-specific manner. (**A**) HEK293T cells were transfected with V5-tagged mouse TRIM25 (V5-mTRIM25), human TRIM25 (V5-hTRIM25) or chicken TRIM25 (V5-chTRIM25) together with NS1-A/California/04/09 (Cal04) (left), or NS1-PR8, NS1-A/Hong Kong/156 (HK156), NS1-A/Swine Texas/98 (SwTx98), or NS1-Cal04 (right panel). Empty vector and V5-tagged human TRIM21 were used as negative controls. Whole-cell lysates (WCLs) of HEK293T cells were subjected to immunoprecipitation (IP) using anti-V5 antibody, followed by immunoblotting (IB) with anti-NS1. (**B–E**) TRIM25 is important for virus-induced IFN-β production in chicken cells. Chicken LMH cells were transiently transfected with non-silencing control siRNA (si.C), or with a siRNA specific for chicken TRIM25 (si.chTRIM25). At 40 h posttransfection, cells were infected with recombinant influenza viruses expressing the NS1 RNA-binding mutant R38A/K41A (**C**), or NS1 from PR8, SwTx98, or HK156 (**D**) at an MOI of 0.1 for 24 h. Knockdown was confirmed by determining chTRIM25 mRNA levels (**B**). Furthermore, the IFN-β (**C and D**) and viral M1 (**E**) mRNA levels were assessed by qPCR and the values were normalized to chicken GAPDH. (**F**) IFN induction and viral replication in murine (MEF) cells. 12 hours postinfection, IFN-β (far left panel) and viral M1 (right panel) mRNA levels were assessed by qPCR from MEFs infected with the different NS1 recombinant viruses at an MOI of 2. Values were normalized to mouse actin. IFN protein was quantified by VSV-GFP bioassay on L929 cells treated with 2-fold dilutions of post-influenza virus supernatants from MEF cells (left panel). Relative fluorescence values reported on the y-axis represent the levels of VSV-GFP replication. High IFN concentrations in the supernatants correspond to low levels of VSV-GFP replication (low fluorescence values). Each sample was assayed in triplicate, and results are representative of two independent experiments. Supernatants were assayed for progeny virus yields 12 h postinfection in standard plaque titrations (far right panel). Virus yields are depicted in PFU/ml. *p<0.05; ***p<0.001.

Consistent with our previous results [38], co-immunoprecipitation (Co-IP) studies in transfected HEK293T cells showed that all tested NS1 proteins efficiently interacted with human TRIM25 (hTRIM25) (Figure 1A). Furthermore, chicken TRIM25 (chTRIM25) bound strongly to NS1-HK156, whereas only a weak or no interaction with chTRIM25 was observed for NS1 from PR8 or Cal04 and SwTx98, respectively (Figure 1A). Moreover, none of the NS1 proteins interacted with mouse TRIM25 (mTRIM25).

It has been suggested that chicken lacks a functional RIG-I gene [39]. We thus asked whether chTRIM25 is important for IFN-β induction upon IAV infection, and further tested if the chTRIM25-NS1 interaction is functional. To this end, we silenced endogenous TRIM25 in chicken LMH cells and measured IFN-β mRNA expression upon viral infection (Figures 1B–1D). Specifically, we used recombinant A/PR/8/34 viruses expressing NS1 proteins from PR8, SwTx98, and HK156, which showed differential binding to chTRIM25, as well as a PR8 recombinant virus expressing the NS1 mutant R38A/K41A, known to be deficient in IFN antagonism due to its abolished dsRNA and hTRIM25 binding ability [10], [38], [41]. Approximately 87% knockdown of chTRIM25 mRNA levels was achieved as determined by quantitative PCR (qPCR) (Figure 1B). Compared to mock-infected control cells, IFN-β mRNA was highly up-regulated (∼1500-fold) upon infection with the NS1 mutant R38A/K41A virus (Figure 1C). This IFN-β induction was significantly reduced in TRIM25-knockdown LMH cells, suggesting that TRIM25 is indeed functional in chicken cells. In addition, IFN-β induction upon infection with PR8, SwTx98 and HK156 recombinant viruses inversely correlated with the ability of their NS1 proteins to bind chTRIM25 (Figure 1D). While PR8 and HK156 were poor inducers of IFN-β mRNA, infection with SwTx98 virus, whose NS1 protein did not bind chTRIM25, resulted in ∼15-fold IFN-β induction. Furthermore, the IFN-β induction by SwTx98 virus was reduced to almost basal levels in TRIM25-knockdown cells, indicating that this virus induces IFN-β gene expression in chicken cells by a mechanism mostly dependent on TRIM25 (Figure 1D). To exclude that the low levels of IFN production induced by the PR8 and HK156 viruses are due to inefficient replication in LMH cells, we measured the mRNA levels of viral M1 by qPCR (Figure 1E). All viruses replicated well, and although only minor differences were observed, the levels of M1 mRNA inversely correlated with the IFN-β inducing activity of these viruses. This suggests that TRIM25 also plays an important role in establishing an antiviral response in chicken cells.

We next tested whether the inability of the NS1 proteins to bind mTRIM25 correlates with their IFN-inducing activities in mouse cells. For this, we infected murine embryonic fibroblasts (MEF) with the different NS1 recombinant viruses. IFN-β mRNA induction was then determined by qPCR, and IFN protein levels were measured by bioassay (Figure 1F, left panels). Surprisingly, although none of the NS1 proteins bound to mTRIM25 (Figure 1A), all NS1 recombinant viruses induced very low amounts of IFN in MEFs as compared to the R38A/K41A mutant virus, suggesting that NS1 does not require binding to TRIM25 for suppressing IFN induction in murine cells. Binding of NS1 to CPSF30 could also not account for this inhibitory activity in MEFs, as all of these NS1 proteins are deficient in inhibiting CPSF30 and thus general gene expression [40]. Furthermore, all viruses replicated to similar levels in the MEF cells as determined by M1 mRNA and virus titrations (Figure 1F, right panels).

**Figure 2:**
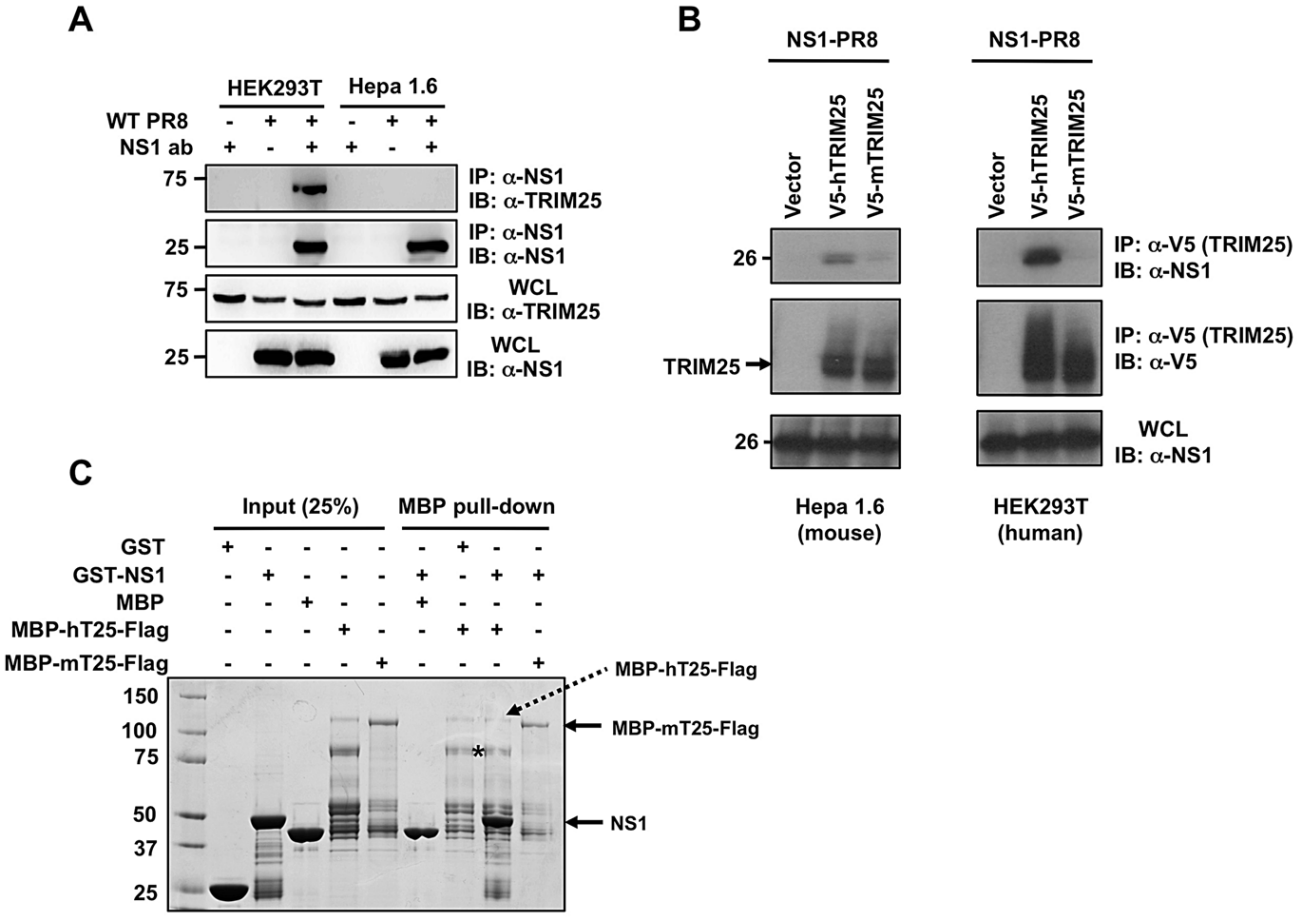
Influenza A Virus NS1 does not interact with mouse TRIM25. (**A**) WCLs of human (HEK293T) or mouse (Hepa1.6) cells, that had been mock-treated or infected with PR8 virus at MOI 2 for 18 h, were subjected to IP with or without anti-NS1 antibody, followed by IB with anti-TRIM25. Expression of NS1 and endogenous TRIM25 was determined by IB with anti-NS1 or anti-TRIM25 antibody. (**B**) Mouse Hepa1.6 or human HEK293T cells were transfected with empty vector, V5-tagged mouse TRIM25 (V5-mTRIM25), or V5-tagged human TRIM25 (V5-hTRIM25), together with NS1-PR8. At 30 h posttransfection, WCLs were prepared and subjected to IP with anti-V5 antibody, followed by immunoblotting with anti-NS1 or anti-V5. (**C**) *In vitro* binding of NS1 with TRIM25. Maltose-binding protein (MBP), GST, and the recombinant fusion proteins MBP-human TRIM25-Flag (MBP-hTRIM25-Flag), MBP-mouse TRIM25-Flag (MBP-mTRIM25-Flag), and GST-NS1 (PR8) were purified from bacteria. Purified MBP-hTRIM25-Flag or MBP-mTRIM25-Flag was incubated with GST-NS1. As controls for binding, purified MBP was incubated with GST-NS1, and MBP-hTRIM25-Flag was incubated with GST. Protein complexes were then precipitated with Amylose agarose gel, and precipitates resolved by SDS-PAGE, followed by Coomassie staining. Asterisk indicates the main degradation product of MBP-hTRIM25-Flag.

Mice are frequently used as a model to study influenza pathogenesis. It is therefore important to understand the detailed mechanisms by which influenza virus escapes the immune response in this particular host. Thus, we first sought to confirm the lack of NS1 interaction with mTRIM25. To rule out that the inability of NS1 to bind to mTRIM25 was due to the absence of a co-factor required for NS1-TRIM25-interaction in human cells, we tested the binding of NS1-PR8 to endogenous TRIM25 in PR8-infected human HEK293T and murine Hepa1.6 cells (Figure 2A). NS1 efficiently interacted with endogenous TRIM25 in HEK293T cells, whereas it did not bind to endogenous TRIM25 in Hepa1.6 cells (Figure 2A). We also observed an efficient interaction of ectopically expressed NS1 with exogenous hTRIM25, but not with mTRIM25, in HEK293T and Hepa1.6 cells (Figure 2B). In line with this, bacterially purified Glutathione-*S*-transferase (GST)-NS1 fusion protein bound to hTRIM25, but not to mTRIM25, in an *in vitro* binding assay (Figure 2C).

**Figure 3:**
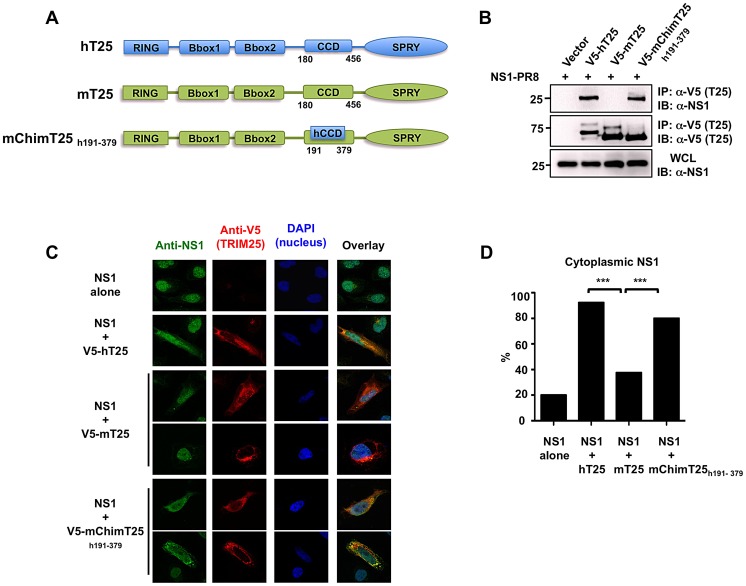
Chimeric mouse TRIM25 containing the CCD of human TRIM25 recovers NS1 binding. (**A**) Schematic representations of human TRIM25 (hT25), mouse TRIM25 (mT25) and the mouse/human TRIM25 chimera (mChimT25_h191–379_). Numbers indicate amino acid. (**B**) WCLs of HEK293T cells, that had been transfected with empty vector, V5-hTRIM25, V5-mTRIM25 or V5-mChimT25_h191–379_ together with NS1-PR8, were subjected to IP with anti-V5 antibody, followed by IB with anti-NS1. (**C**) Localization of NS1-PR8 in HeLa cells determined by confocal microscopy. At 30 h posttransfection with NS1-PR8 alone, NS1-PR8 together with V5-hTRIM25, V5-mTRIM25, or V5-mChimT25_h191–379_, HeLa cells were stained with anti-NS1 (green), anti-V5 (red) and DAPI (nucleus, blue). (**D**) Quantification of cytoplasmic NS1 localization from (C). From three independent experiments, 50 cells with expression of NS1 alone or NS1 together with hTRIM25, mTRIM25 or mChimT25_h191–379_, respectively, were counted and the percentage of cells with cytoplasmic NS1 is shown, ***p<0.001 by Fisher's exact test.

The CCDs of human and mouse TRIM25 proteins share only 56% identity at the amino acid level (aa 180–450) with major differences between aa 350–400. To test if these amino acid changes in TRIM25 CCD are responsible for the difference in NS1 binding we generated a human/mouse chimeric TRIM25 protein containing amino acids 191–379 of hTRIM25 (mChimT25_h191–379_) and analyzed its ability to interact with NS1-PR8 (Figures 3A and 3B). Co-IP experiments showed that while mTRIM25 did not interact with NS1, hTRIM25 and the mChimT25_h191–379_ efficiently bound NS1 (Figure 3B).

As previously reported [38], overexpressed NS1 localizes primarily to the nucleus with a minor cytoplasmic component; however, co-expressed hTRIM25 led to a marked increase of NS1 cytoplasmic localization, indicative of hTRIM25-NS1 interaction. As shown in Figures 3C and 3D, ectopic expression of hTRIM25 or mChimT25_h191–379_ markedly re-localized NS1 to the cytoplasm, where they extensively co-localized (92% and 80% of cells with cytoplasmic NS1, respectively). In contrast, co-expression of mTRIM25 that did not interact with NS1 showed only a minor effect on NS1 sub-cellular localization (Figures 3C and 3D). These results collectively demonstrate that amino acid changes in the CCD region of mouse TRIM25 are responsible for the loss of interaction with NS1.

### NS1-PR8 Inhibits Mouse RIG-I Ubiquitination and Downstream IFN Induction in a TRIM25-independent Manner

**Figure 4:**
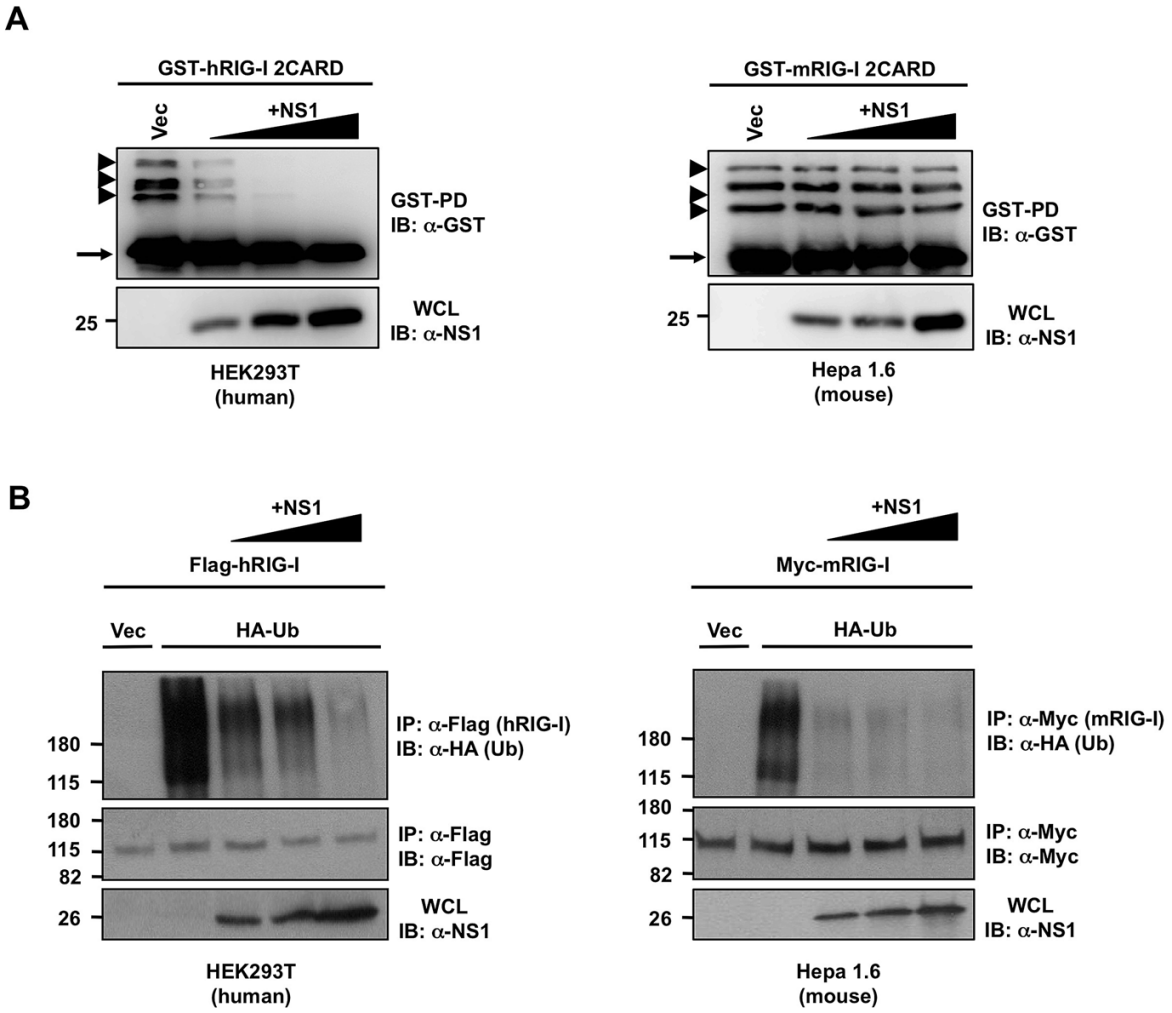
NS1 inhibits the ubiquitination of full-length RIG-I but not that of RIG-I 2CARD in murine cells. (**A**) Human HEK293T (**left**) or mouse Hepa 1.6 (**right**) cells were transfected with human GST-RIG-I 2CARD (GST-hRIG-I 2CARD) (**left**) or mouse GST-RIG-I 2CARD (GST-mRIG-I 2CARD) (**right**) and increasing amounts of NS1-PR8 (**left and right**). At 40 h posttransfection, WCLs were subjected to GST-pulldown (GST-PD), followed by IB with anti-GST antibody. Arrow heads indicate ubiquitinated bands. Arrow indicates non-ubiquitinated 2CARD. (**B**) Human HEK293T (**left**) or mouse Hepa1.6 (**right**) cells were transfected with Flag-tagged human RIG-I (Flag-hRIG-I) or Myc-tagged mouse RIG-I (Myc-mRIG-I) respectively, together with empty vector or HA-ubiquitin and increasing amounts of NS1-PR8. At 24 h posttransfection, cells were infected with SeV (10 HA units/ml) for 10 h. WCLs were subjected to IP with anti-Flag (**left**) or anti-Myc (**right**), followed by IB with the indicated antibodies.

Influenza A viruses lacking functional NS1 proteins induce a robust IFN response, and thus replicate poorly in immunocompetent mice, indicating that NS1 inhibits induction of IFN in mice [42]. However, while in human cells NS1 interacts with human TRIM25 for inhibition of RIG-I ubiquitination and activation [38], the same mechanism cannot apply to mouse cells, as NS1 does not interact with mouse TRIM25. In order to gain more insights on the impact of NS1 on mouse RIG-I activation we examined the effect of NS1 on the ubiquitination of human or mouse RIG-I 2CARD in human HEK293T or mouse Hepa1.6 cells (Figure 4A).

Consistent with our previous results [38], NS1-PR8 potently inhibited the ubiquitination of GST-fused human RIG-I 2CARD (GST-hRIG-I 2CARD) in HEK293T cells in a dose-dependent manner. In striking contrast, NS1 did not block the ubiquitination of mouse GST-RIG-I 2CARD (GST-mRIG-I 2CARD) in Hepa1.6 cells (Figure 4A). Furthermore, we analyzed the effect of NS1 on the Sendai virus (SeV)-induced ubiquitination of full-length RIG-I in human HEK293T and murine Hepa1.6 cells (Figure 4B). Interestingly, exogenously expressed NS1-PR8 effectively suppressed the ubiquitination of full-length RIG-I in a dose-dependent manner in both cell lines (Figure 4B). These results collectively suggest that while NS1 efficiently inhibits the CARD ubiquitination of human RIG-I, it suppresses the ubiquitination of mouse RIG-I by acting on its C-terminal region.

**Figure 5:**
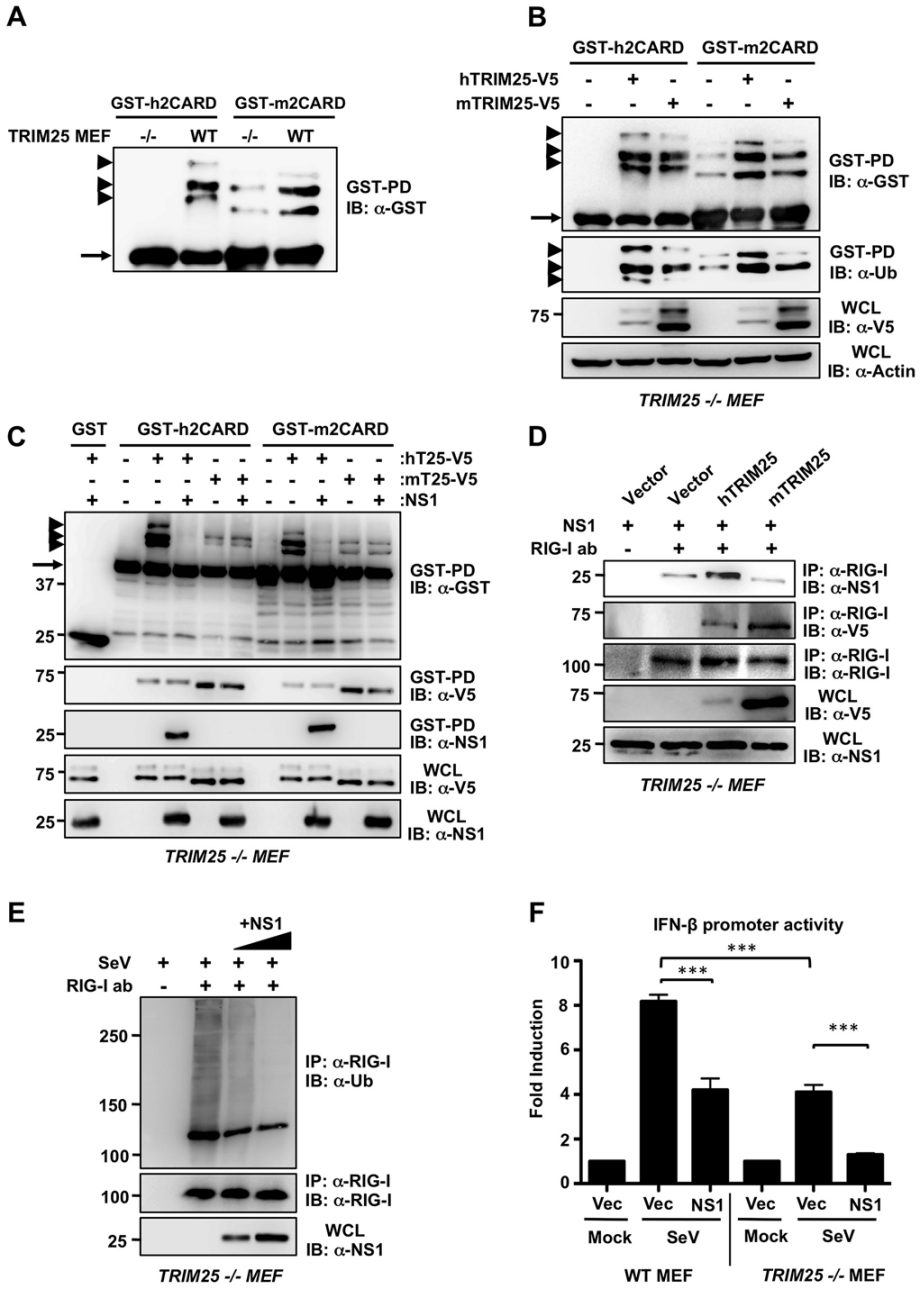
NS1-mediated suppression of RIG-I ubiquitination and IFN induction in mouse is TRIM25-independent. (**A**) Ubiquitination of the RIG-I CARDs in WT and *TRIM25 −/−* MEF cells. WT and *TRIM25 −/−* MEFs were transfected with human GST-RIG-I 2CARD (GST-h2CARD) or mouse GST-RIG-I 2CARD (GST-m2CARD). At 40 h posttransfection, WCLs were subjected to GST-pulldown (GST-PD), followed by IB with anti-GST antibody. Arrow heads indicate ubiquitinated bands. Arrow indicates non-ubiquitinated 2CARD. (**B**) Ubiquitination of the RIG-I CARDs in *TRIM25 −/−* cells reconstituted with mouse or human TRIM25. At 40 h posttransfection with human or mouse GST-RIG-I 2CARD together with empty vector, V5-tagged hTRIM25 or mTRIM25, WCLs of *TRIM25 −/−* MEFs were subjected to GST-PD, followed by IB with anti-GST or anti-ubiquitin (Ub) antibody. Expression of TRIM25 proteins was determined by IB with anti-V5 antibody. Arrow heads indicate ubiquitinated bands. Arrow indicates non-ubiquitinated 2CARD. (**C**) NS1 inhibits the RIG-I CARD ubiquitination induced by human TRIM25 but not by mouse TRIM25. *TRIM25 −/−* MEFs were transfected with GST, human GST-RIG-I 2CARD (GST-h2CARD) or mouse GST-RIG-I 2CARD (GST-m2CARD) together with vector, hTRIM25-V5 or mTRIM25-V5 with or without NS1. At 40 h posttransfection, WCLs were subjected to GST-PD, followed by IB with anti-GST for detecting RIG-I CARD ubiquitination, anti-V5 for detecting TRIM25 binding, or anti-NS1 antibody for detecting NS1 binding. Arrow heads indicate ubiquitinated bands. Arrow indicates non-ubiquitinated 2CARD. (**D**) NS1 interacts with mouse RIG-I in a TRIM25-independent manner. *TRIM25 −/−* MEFs were transfected with empty vector, V5-hTRIM25, or V5-mTRIM25 together with NS1-PR8. WCLs were subjected to IP with anti-RIG-I antibody, followed by IB with anti-NS1, anti-V5, or anti-RIG-I antibody. Expression of NS1 and TRIM25 proteins in the WCLs was determined by anti-NS1 and anti-V5 antibody, respectively. (**E**) NS1 inhibits endogenous mouse RIG-I ubiquitination in a TRIM25-independent manner. *TRIM25 −/−* MEFs were transfected with increasing amounts of NS1-PR8. At 32 h posttransfection, cells were infected with SeV (50 HA units/ml) for 10 h. WCLs were subjected to IP with anti-RIG-I antibody, followed by IB with anti-Ub or anti-RIG-I antibody. (**F**) NS1 inhibits IFN-β induction in murine cells in a TRIM25-independent manner. WT and *TRIM25 −/−* MEFs were transfected with an IFN-β luciferase construct together with empty vector or NS1. At 24 h posttransfection, cells were either mock-treated, or infected with SeV (10 HA units/ml) for 16 h. Samples were then subjected to a dual luciferase assay. Data represent the mean ± SD (n = 3).

Lys172 in the CARDs of human RIG-I (hRIG-I) is essential for RIG-I ubiquitination and activation by TRIM25 [25]. However, this residue is not conserved in mouse RIG-I (mRIG-I) (Supplemental Figure S2), raising the question whether mRIG-I is ubiquitinated by mTRIM25 as previously reported to occur for human RIG-I. Strikingly, ubiquitination of GST-h2CARD was undetectable in *TRIM25 −/−* MEFs, whereas it was robustly ubiquitinated in wild-type (WT) MEFs (Figure 5A). In contrast, GST-m2CARD showed a decreased but still detectable ubiquitination in *TRIM25 −/−* MEFs compared to WT MEFs (Figure 5A). Reconstitution of *TRIM25 −/−* MEFs with mouse or human TRIM25 showed an increase in both human and mouse RIG-I 2CARD ubiquitination (Figure 5B), indicating that mTRIM25 is active and is able to ubiquitinate the mouse RIG-I CARDs under these conditions at a different Lys residue/s. Interestingly, NS1 inhibited the hTRIM25-dependent ubiquitination of human or mouse RIG-I 2CARD but was unable to inhibit mTRIM25-dependent ubiquitination of human or mouse RIG-I 2CARD (Figure 5C). In support of this, NS1 co-immunoprecipitated with RIG-I 2CARD only when hTRIM25 was present, but not in the presence of ectopically expressed mTRIM25 (Figure 5C). Since NS1 potently inhibited the ubiquitination of full-length mRIG-I (Figure 4B), we next tested if NS1-PR8 interacts with endogenous RIG-I in non-complemented *TRIM25 −/−* MEFs, or in *TRIM25 −/−* MEFs reconstituted with either human or mouse TRIM25 (Figure 5D). This showed that NS1 readily co-immunoprecipitated with endogenous RIG-I in *TRIM25 −/−* MEFs, and this interaction was further increased in the presence of exogenous human but not mouse TRIM25 (Figure 5D). Furthermore, exogenously expressed NS1-PR8 strongly suppressed the SeV-induced ubiquitination of endogenous RIG-I in *TRIM25 −/−* cells in a dose-dependent manner (Figure 5E), indicating that NS1 binds to mouse RIG-I and inhibits its ubiquitination in a TRIM25-independent manner. We next determined the ubiquitination of endogenous RIG-I in WT and *TRIM25 −/−* MEFs upon PR8 virus infection (Supplemental Figure S4A). This showed that RIG-I ubiquitination was decreased in *TRIM25 −/−* MEFs compared to WT MEFs. Furthermore, PR8 infection markedly reduced the ubiquitination of endogenous RIG-I in both WT MEFs and *TRIM25 −/−* MEFs (Supplemental Figure S4A).

Finally, we examined the ability of NS1-PR8 to inhibit IFN-β promoter activation in WT MEFs or *TRIM25 −/−* MEFs. As expected, NS1 inhibited SeV-induced IFN-β promoter activation in WT MEFs (Figure 5F). *TRIM25 −/−* MEFs showed a markedly reduced IFN-β promoter activity upon SeV infection as compared to WT MEFs; however there was still detectable IFN-β induction by SeV stimulation. This residual IFN-β induction in *TRIM25 −/−* MEFs was further suppressed by exogenous NS1 (Figure 5F), demonstrating that NS1 inhibits the IFN induction in mouse cells by a TRIM25 independent mechanism.

Collectively, these results demonstrate that human TRIM25 is the major ubiquitin E3 ligase for human RIG-I 2CARD and that NS1 inhibits its activity. Our results further indicate that in mouse cells there is an alternative ubiquitin E3 ligase that cooperates with TRIM25 for full activation of mouse RIG-I, and that this alternative ubiquitin E3 ligase is probably targeted by NS1.

### NS1 Interacts with Mouse Riplet Ubiquitin E3 Ligase

Riplet (also known as RNF135) has recently been shown to be important for IFN production in mice [27] and to ubiquitinate human RIG-I at Lys849 and Lys851 in its C-terminal domain [26]. Since these lysine residues are conserved in mouse RIG-I (Supplemental Figure S3) we hypothesized that NS1 may bind mouse Riplet (mRiplet), thereby suppressing RIG-I ubiquitination and downstream signaling in mouse.

**Figure 6:**
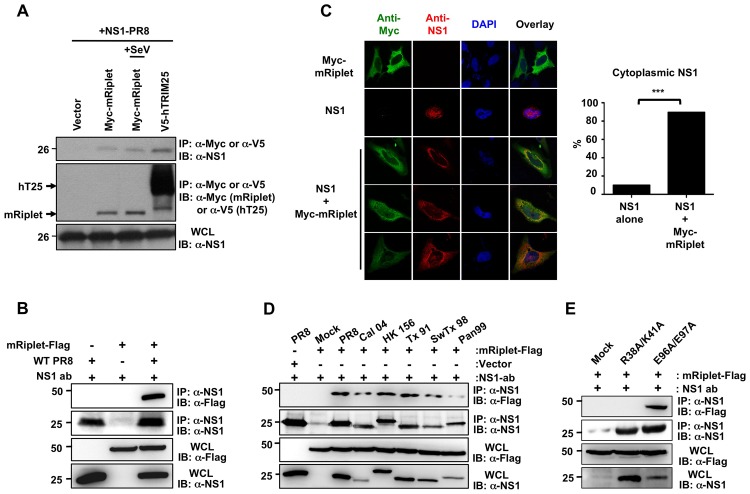
Influenza A Virus NS1 interacts with mouse Riplet. (**A**) At 30 h posttransfection with Myc-tagged mouse Riplet (Myc-mRiplet) together with NS1-PR8, HEK293T cells were mock-treated or infected with SeV (10 HA units/ml) for 10 h. V5-tagged hTRIM25 was co-transfected as positive control. WCLs were subjected to IP with anti-Myc (Riplet) or anti-V5 (TRIM25), followed by IB with anti-NS1 antibody. (**B**) Hepa1.6 cells were transfected with Flag-tagged mouse Riplet. At 30 h posttransfection, cells were either mock-treated or infected with recombinant A/PR/8/34 virus at an MOI of 2. 18 h later, WCLs were subjected to IP with anti-NS1 antibody, followed by immunoblotting using the indicated antibodies. (**C**) Localization of NS1-PR8 and mouse Riplet in HeLa cells determined by confocal microscopy. At 30 h posttransfection with Myc-mRiplet alone, NS1-PR8 alone, or NS1 together with Myc-mRiplet, HeLa cells were stained with anti-Myc (green), anti-NS1 (red), and DAPI (nucleus, blue). Fifty cells from three independent experiments were counted and the percentage of cells with cytoplasmic NS1 is shown, ***p<0.001 by Fisher's exact test. (**D and E**) Hepa1.6 cells were transfected with Flag-tagged mouse Riplet. At 30 h posttransfection, cells were either mock-treated or infected with recombinant A/PR/8/34 virus expressing NS1 PR8, Cal04, HK156, Tx91, SwTx98, or Pan99 at an MOI of 2 (**D**), or R38A/K41A or E96A/E97A NS1 mutant at an MOI of 4 (**E**). 18 h later, WCLs were subjected to IP with anti-NS1 antibody, followed by immunoblotting using the indicated antibodies.

As shown in Figure 6A, NS1-PR8 readily bound exogenously expressed mRiplet in HEK293T cells in the presence or absence of SeV infection. We also observed an efficient interaction of NS1 with mRiplet in Hepa1.6 cells infected with the PR8 strain (Figure 6B). Immunofluorescence assays further showed that, as seen with human TRIM25, co-expressed mRiplet re-localized NS1 from the nucleus to the cytoplasm; approximately 90% of the cells exhibited primarily cytoplasmic NS1 (Figure 6C). Furthermore, exogenously expressed NS1 of Cal04 and HK156 strains also efficiently interacted with mRiplet in murine Hepa1.6 cells (Supplemental Figure S4B). In addition, Hepa1.6 cells infected with various recombinant A/PR/8/34 viruses expressing NS1 proteins from PR8, Cal04, HK156, Tx91, SwTx98, and Pan99, also showed an interaction between NS1 and mRiplet (Figure 6D), indicating that this effect is not exclusive of the NS1 protein of the mouse-adapted PR8 strain. Finally, the NS1 E96A/E97A mutant that lacks the ability to bind human TRIM25 [38] was also able to interact with mRiplet in transfected and infected cells, whereas the NS1 R38A/K41A mutant, lacking both dsRNA and hTRIM25 binding, did not bind to mRiplet under the same conditions (Figure 6E and Supplemental Figure S4C).

### NS1 Antagonizes the Riplet-dependent RIG-I Ubiquitination and IFN Induction in Murine Cells

**Figure 7:**
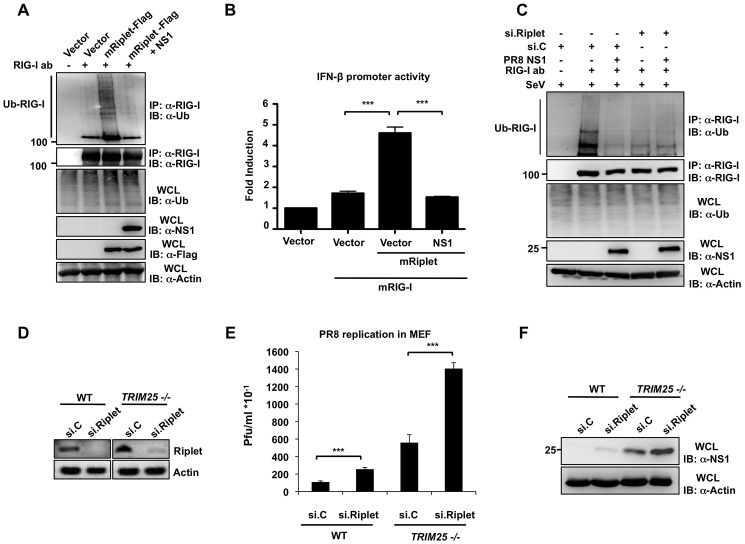
NS1 inhibits the Riplet-dependent RIG-I ubiquitination and IFN induction in murine cells. (**A**) Mouse Hepa 1.6 cells were transfected with vector or Flag-tagged mRiplet with or without NS1-PR8. WCLs were subjected to IP with RIG-I antibody, followed by IB with anti-Ub or anti-RIG-I antibody. Expression of mRiplet, NS1, and ubiquitin (Ub) was determined in the WCLs by IB with anti-Flag, anti-NS1 or anti-Ub antibody. (**B**) Mouse Hepa1.6 cells were transfected with IFN-β luciferase reporter plasmid together with empty vector or mRIG-I together with or without mRiplet and NS1-PR8. At 24 h posttransfection, cells were lysed and subjected to luciferase assay. Data shown is representative of 3 independent experiments and depicted is the mean ± SD (n = 3). (**C**) Influenza NS1 protein specifically inhibits the Riplet-dependent ubiquitination of mouse RIG-I. Hepa1.6 cells were transiently transfected with non-silencing control siRNA (si.C), or with a siRNA specific for mouse Riplet (si.Riplet) together with empty vector or NS1-PR8. At 24 h posttransfection, cells were infected with SeV (50 HA units/ml) for 22 h. WCLs were used for IP with anti-RIG-I antibody, followed by IB with anti-Ub or anti-RIG-I antibody. (**D–F**) Knockdown of endogenous Riplet in mouse embryonic fibroblasts enhances influenza A virus replication. WT or *TRIM25 −/−* MEFs were transfected with non-silencing control siRNA (si.C), or with a siRNA specific for mouse Riplet (si.Riplet). At 30 h posttransfection, cells were infected with recombinant A/PR/8/34 WT virus (MOI 0.1). Knockdown of endogenous Riplet was confirmed by RT-PCR (**D**). Supernatants were assayed for progeny virus yields 24 h postinfection in standard plaque titrations (**E**). Virus yields are depicted in Pfu/ml. The results of three independent experiments are shown. Furthermore, viral NS1 protein expression was determined in the WCLs of infected cells (**F**).

We next asked whether NS1 specifically suppresses the Riplet-mediated ubiquitination of RIG-I in murine cells. For this, we examined the endogenous RIG-I ubiquitination in murine Hepa1.6 cells that were transfected with empty vector or Flag-mRiplet together with or without NS1-PR8 (Figure 7A). This showed that exogenous expression of mRiplet markedly enhanced the ubiquitination of endogenous RIG-I, and this ubiquitination was potently suppressed by co-expression of NS1 (Figure 7A). Consistent with this, ectopic expression of mRiplet led to a 4.6-fold increase of mRIG-I-induced IFN-β promoter activation, and this RIG-I activation was inhibited almost to basal levels by co-expressed NS1 (Figure 7B). Finally, we sought to determine the role of endogenous Riplet in mouse RIG-I ubiquitination, and also addressed whether NS1 specifically antagonizes the mRiplet-induced RIG-I ubiquitination. For this, we examined the ubiquitination of RIG-I in Hepa1.6 cells in which endogenous Riplet was silenced using specific siRNA (Figure 7C). 78% knockdown efficiency was achieved as determined by RT-PCR analysis compared to non-silencing control siRNA (data not shown). Specific knockdown of endogenous Riplet strongly decreased endogenous RIG-I ubiquitination (Figure 7C). Furthermore, co-expressed NS1 efficiently blocked the ubiquitination of RIG-I in cells transfected with non-silencing control siRNA, while NS1 had no effect on the residual ubiquitination of RIG-I observed in Riplet-knockdown cells (Figure 7C). These results collectively demonstrate that Riplet is an important ubiquitin E3 ligase for mouse RIG-I, and that NS1 specifically targets Riplet to inhibit RIG-I ubiquitination in murine cells.

To further demonstrate that Riplet contributes to the establishment of an efficient antiviral response against influenza virus, we silenced endogenous Riplet in WT or *TRIM25 −/−* MEFs using specific siRNA, and assessed influenza PR8 virus replication (Figures 7D–7F). The knockdown of mRiplet was confirmed by RT-PCR (Figure 7D). Infection of *TRIM25 −/−* cells with PR8 virus resulted in higher plaque forming units (PFU) as compared with WT MEF cells, underscoring the important role of TRIM25 for an efficient antiviral response in mouse. Furthermore, siRNA-mediated knockdown of endogenous Riplet in both WT and *TRIM25 −/−* cells yielded significantly higher levels of viral replication compared to transfection of non-silencing siRNA, with the strongest effect observed in *TRIM25 −/−* MEFs in which Riplet was silenced (Figure 7E). The increased virus replication in Riplet-knockdown MEFs correlated with increased NS1 protein levels in these cells (Figure 7F). Taken together, these data demonstrate that both Riplet and TRIM25 contribute to an effective antiviral response in mouse.

### The NS1 Proteins from Human Influenza Viruses Bind and Inhibit Human Riplet

**Figure 8:**
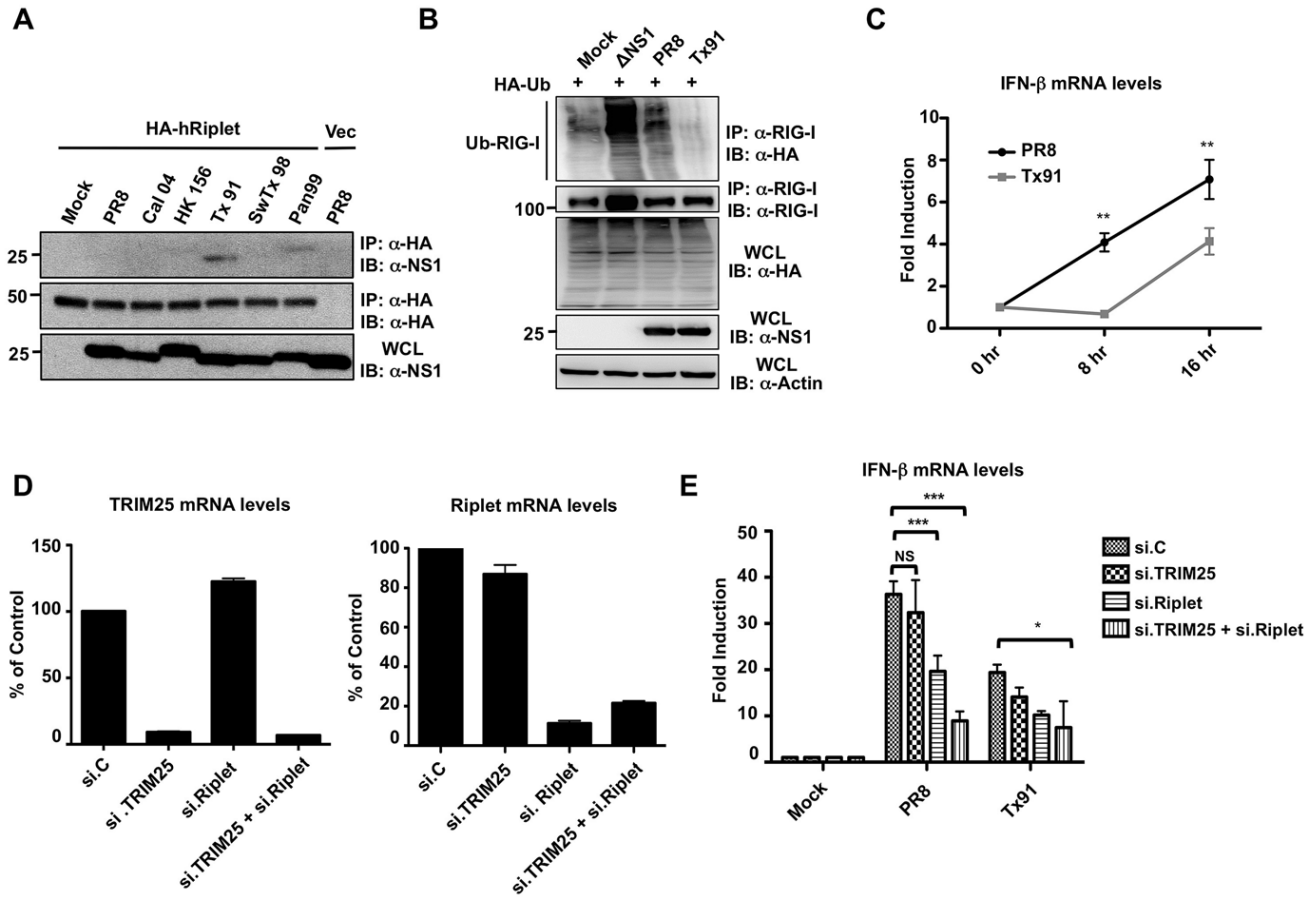
NS1 proteins from human influenza strains bind and inhibit human Riplet. (**A**) HEK293T cells were transfected with empty vector or HA-tagged human Riplet (HA-hRiplet). At 30 h posttransfection, cells were either mock-treated, or infected with the indicated recombinant A/PR/8/34 viruses at an MOI of 2. 18 h later, WCLs were subjected to IP with anti-HA antibody, followed by immunoblotting using the indicated antibodies. (**B**) Tx91 recombinant virus suppresses the endogenous RIG-I ubiquitination more potently than PR8 virus. HEK293T cells, that had been transfected with HA-tagged ubiquitin, were either mock-treated, or infected with ΔNS1 PR8, PR8 WT, or Tx91-NS1 recombinant virus at an MOI of 2 for 18 h. WCLs were subjected to IP with anti-RIG-I antibody, followed by IB with anti-HA or anti-RIG-I antibody. Expression of HA-ubiquitin, viral NS1, and Actin was further determined in the WCLs. (**C**) A549 cells were infected with PR8-NS1 or Tx91-NS1 recombinant virus at an MOI of 0.1. Cells were collected at the indicated time points and IFN-β mRNA was measured by qPCR. (**D and E**) A549 cells were transiently transfected with non-silencing control siRNA (si.C), or with siRNA specific for TRIM25 (si.TRIM25), Riplet (si.Riplet), or both. At 40 h posttransfection, cells were infected with PR8 WT or Tx91 recombinant virus at an MOI of 2 for 30 h. The mRNA levels of TRIM25 and Riplet were measured by qPCR for analyzing their knockdown efficiency (**D**). Furthermore, IFN-β mRNA levels were assessed by qPCR (**E**). Results are triplicates from 3 independent experiments. NS; statistically non-significant.

Mice are not a natural host of influenza viruses, but the fact that all NS1 proteins tested interacted with mouse Riplet, including non-mouse adapted NS1 proteins (avian or swine) suggests that this is a conserved evasion mechanism across influenza viruses. Therefore, we tested if human Riplet interacts with the different NS1 proteins in the context of viral infection. Interestingly, the NS1 from human influenza viruses (Tx91 and Pan99) interacted with human Riplet (Figure 8A), suggesting that the capacity of NS1 binding to mouse Riplet might reflect an interaction of NS1 with Riplet derived from its specific host. To further demonstrate that the interaction of NS1 with human Riplet is biologically relevant, we compared the capacity of Tx91-NS1 and PR8-NS1 recombinant viruses to inhibit the ubiquitination of human RIG-I (Figure 8B). We reasoned that Tx91-NS1 that binds both hTRIM25 and hRiplet, should be more efficient in inhibiting RIG-I ubiquitination (and IFN induction) than PR8-NS1 shown to bind only hTRIM25. As predicted, Tx91-NS1 recombinant virus was more efficient in inhibiting human RIG-I ubiquitination as compared to PR8-NS1 virus (Figure 8B). Accordingly, Tx91-NS1 recombinant virus induced significantly lower levels of IFN-β mRNA as compared to PR8 virus (Figure 8C). Finally, we analyzed IFN-β production in PR8- and Tx91-infected A549 cells upon knockdown of TRIM25, Riplet or both (Figures 8D and 8E). siRNA-mediated knockdown reduced TRIM25 and Riplet mRNA levels by approximately 90% and 80%, respectively. Furthermore, the knockdown was highly specific, as targeting TRIM25 did not reduce Riplet mRNA levels and *vice-versa* (Figure 8D). Silencing of endogenous TRIM25 in PR8-infected cells resulted in a slight but not significant decrease of IFN-β induction as compared to cells transfected with non-silencing control siRNA (Figure 8E). In contrast, IFN induction by PR8 was significantly reduced in Riplet-knockdown cells as compared to controls, and almost to the same extent as the levels induced by the Tx91 virus in control cells. These results suggest that while PR8 inhibits IFN induction by targeting TRIM25, the residual IFN induction (not inhibited by the virus) depends mostly on Riplet. Conversely, knockdown of either TRIM25 or Riplet did not have a significant effect on IFN induction in Tx91-infected cells. A significant reduction in IFN-β mRNA levels was only observed in cells in which both TRIM25 and Riplet were silenced (Figure 8E). Collectively, these results indicate that both TRIM25 and Riplet are important for IFN-β production in human cells upon influenza virus infection. Furthermore, while PR8-NS1 mainly targets TRIM25 in human cells, Tx91-NS1 interacts with and suppresses both TRIM25 and Riplet for the inhibition of IFN production in human cells.

## Discussion

**Figure 9:**
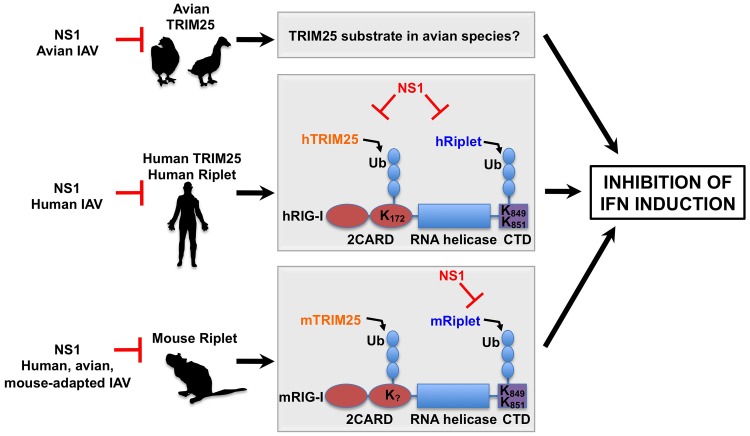
Proposed model of the species-specific inhibition of RIG-I by influenza A virus NS1 protein. The NS1 proteins of avian influenza viruses bind to chicken TRIM25, thereby suppressing IFN induction. The substrate of TRIM25 in avian cells has yet to be determined. The NS1 proteins from human influenza viruses bind to and inhibit both human TRIM25 and human Riplet, thereby suppressing the ubiquitination of the RIG-I CARD and CTD. The NS1 proteins from avian, human and mouse-adapted influenza viruses block Riplet but not TRIM25 for the inhibition of RIG-I signaling in mouse cells.

In this study we investigated the mechanisms by which the NS1 protein of influenza A viruses affects the TRIM25/RIG-I-mediated type-I IFN response in different host species (Figure 9). We demonstrated that NS1 proteins from avian and mammalian isolates are capable of binding to human, but not mouse TRIM25. In mouse cells, NS1 protein inhibits RIG-I ubiquitination and downstream IFN promoter activation primarily by a mechanism that involves binding to and inhibiting the ubiquitin E3 ligase Riplet. This mechanism thus clearly differs from the previously described mechanism for NS1 antagonism of TRIM25 in human cells. As mice are not natural hosts of influenza virus infection, we reasoned that the ability of influenza virus NS1 to inhibit mouse Riplet most likely reflects its inherent ability to inhibit Riplet from other animal species. Indeed, our study showed that the NS1 protein of human viruses efficiently bound and suppressed human Riplet, indicating that specifically human viruses have evolved to inhibit both TRIM25 and Riplet for the suppression of RIG-I antiviral activity in human cells. Interestingly, Riplet is highly similar to TRIM25 sharing 60.8% sequence homology. Like TRIM25, Riplet contains an N-terminal RING domain and a C-terminal SPRY domain. However, in contrast to TRIM25, Riplet does not contain a B-box domain and thus it does not belong to the TRIM protein family [26]. Importantly, bioinformatic analysis also predicted the presence of a central CCD in Riplet, with structural similarities to the TRIM25 CCD (data not shown). Further studies are needed to identify the precise binding site of NS1 in Riplet, and whether this interaction requires the Riplet CCD.

Furthermore, our interaction studies showed that an avian NS1 protein (HK156) strongly and preferentially interacted with chicken TRIM25. Although RIG-I has been reported to be absent in chicken cells [39], the fact that avian NS1 efficiently interacted with chicken TRIM25 and that the HK156 virus was a poor inducer of IFN in chicken cells, suggests that this interaction is likely to be functional. In addition, we observed higher levels of IFN induction in chicken cells by SwTx98 recombinant virus correlating with the inability of its NS1 protein to bind chicken TRIM25. Knockdown of TRIM25 in chicken cells resulted in reduced IFN-β induction in infected cells, further evidencing that TRIM25 is active and that NS1 targets its function in chicken cells. Importantly, there are no Riplet sequences reported for the chicken or duck genomes, suggesting that influenza A viruses that are maintained in avian species have adapted to coexist with their host by targeting TRIM25, but not Riplet (Figure 9). Further studies will be required to elucidate the target of TRIM25 in chicken, and whether the same principles apply to the natural avian reservoir of influenza viruses, migratory ducks, which are known to have RIG-I [39].

By studying RIG-I ubiquitination and signal-transducing activity in murine cells, we have also uncovered commonalities and differences between human and mouse RIG-I regulation. Although Lys172, the critical residue for RIG-I ubiquitination in human [25], is not conserved in mouse, we observed a robust ubiquitination of mouse RIG-I 2CARD, suggesting that other lysine residues in the mouse RIG-I CARDs are ubiquitinated. Furthermore, our studies using *TRIM25 −/−* cells indicate that the ubiquitination of mouse RIG-I 2CARD, in contrast to that of human RIG-I 2CARD, is less affected in the absence of TRIM25, indicating that in addition to TRIM25 another ubiquitin E3 ligase mediates RIG-I 2CARD ubiquitination in mouse. The ubiquitin E3 ligase Riplet has been reported to ubiquitinate RIG-I, thereby leading to its optimal activation [26], [27], [43]. However, while Riplet has been shown to ubiquitinate Lys849 and Lys851 in the CTD of RIG-I, the role of Riplet for RIG-I CARD ubiquitination has been a subject of debate [26], [27], [43]. Our data suggests that, at least in the mouse system, Riplet is unlikely to ubiquitinate the 2CARD, because NS1 was unable to inhibit mouse RIG-I 2CARD ubiquitination. Therefore, in addition to TRIM25 there may be an additional ubiquitin E3 ligase in mice that ubiquitinates the RIG-I N-terminal CARDs, and this E3 ligase is most likely not targeted by NS1. Nevertheless, our studies clearly showed that Riplet plays a crucial role for RIG-I ubiquitination-dependent activation in mouse, most likely by ubiquitinating the C-terminal region of RIG-I. Additional work will be needed to further define the roles and contributions of these ubiquitin E3 ligases for RIG-I activation in mouse.

For our interaction studies, we used NS1 proteins of influenza viruses that are not able to bind CPSF30. This facilitates the interpretation of the results, as their expression is not associated with inhibition of general host protein expression. However, we have also shown that the NS1 proteins of the Tx91 and Pan99 strains, which were previously shown to interact with CPSF30 [40], are also able to interact with human and mouse Riplet in infected cells (Figures 6D and 8A). This suggests that NS1 binding to Riplet or CPSF30 is not mutually exclusive.

The NS1 mutant E96A/E97A was previously reported to lack human TRIM25 binding but retained dsRNA binding [38]. Our results showed that the E96A/E97A NS1 mutant, but not the R38A/K41A mutant, efficiently interacted with mouse Riplet, suggesting that the region in NS1 important for dsRNA binding is needed for interaction with mouse Riplet. Alternatively, the interaction between NS1 and mouse Riplet could be mediated by viral RNA.

In our previous study [38], we also showed that the PR8 recombinant virus carrying the E96A/E97A NS1 mutant was able to induce IFN in WT and *TRIM25 +/−* MEFs, but it lost this IFN-inducing capacity in *TRIM25 −/−* cells. In contrast, the R38A/K41A NS1 recombinant virus and a PR8 virus lacking NS1 (ΔNS1 PR8 virus) retained some IFN-inducing capacity in *TRIM25 −/−* cells [38]. Although we cannot completely rule out that in our experiments a low affinity interaction between NS1 and mouse TRIM25 undetectable in our system occurs, the fact that the E96A/E97A mutant virus was able to repress the residual IFN induction in *TRIM25 −/−* cells [38] implies that this virus can, at least in part, block IFN induction in mouse in a TRIM25-independent manner. This probably occurs by binding dsRNA and/or by interacting with other host factors including mouse Riplet.

Our work raises an important question: why does NS1 target Riplet instead of TRIM25 in mouse cells? Our results combined with previous studies [25]–[27], [43] suggest that although both TRIM25 and Riplet contribute to optimal activation of both mouse and human RIG-I, their requirement might be different in human and mouse cells. Our experiments in *TRIM25 −/−* cells showed that whereas human RIG-I 2CARD ubiquitination is undetectable in *TRIM25 −/−* cells, mouse RIG-I 2CARD showed residual ubiquitination under these conditions. In addition, knockdown of endogenous Riplet in mouse cells strongly decreased RIG-I full-length ubiquitination. Taken together, this suggests that while TRIM25 is the primary E3 ligase for human RIG-I ubiquitination, in mouse other ubiquitin E3 ligases including Riplet may play a crucial role for RIG-I ubiquitination-dependent activation. Under this scenario, in mouse cells NS1 must target Riplet for efficient inhibition of RIG-I signaling (Figure 9). The mechanistic differences by how influenza virus NS1 inhibits the RIG-I pathway in human and mouse cells indicate that caution should be used to appropriately interpret future experiments on host responses during influenza virus infection using the mouse model.

Our study also identified two NS1 proteins from human viruses (Tx91 and Pan99) which efficiently bound to human Riplet. Furthermore, a recombinant virus expressing the Tx91 NS1 protein was more efficient in inhibiting endogenous RIG-I ubiquitination and IFN induction in human cells as compared to PR8 WT virus, whose NS1 protein does not bind human Riplet. These results are in agreement with the high capacity of Tx91 virus to inhibit the antiviral state in human dendritic cells [44]. Combined with our knockdown analyses in A549 cells (Figures 8D and 8E), this suggests that human viruses such as Tx91 have evolved to interact with both TRIM25 and Riplet in human cells for the comprehensive inhibition of the RIG-I-induced IFN response (Figure 9).

Finally, although we have used representative strains from different hosts, we cannot exclude that not all strains from a specific host have the observed specificity of antagonism. Future studies are needed to test NS1 proteins of various different strains for their ability to inhibit TRIM25 and Riplet from multiple hosts, and whether this correlates with the ability of these viruses to suppress the IFN response in the respective host.

Taken together, our work suggests that influenza viruses can readily adapt to circumvent the type-I IFN system in different host species by targeting host-specific proteins involved in IFN induction. It stresses the importance of host-specific signaling pathways and immune responses when choosing animal models for studying influenza pathogenicity. Understanding the changes that viruses undergo during adaptation may provide us with better tools to predict potential future pandemics and develop vaccines and antivirals.

## Materials and Methods

### Cell Culture and Transfection

HEK293T (human), HeLa (human), A549 (human), Hepa1.6 (mouse), Vero, and L929 cells were purchased from the American Type Culture Collection (ATCC). WT and *TRIM25 −/−* MEFs were previously described [25]. All cells were maintained in Dulbecco's Modified Eagle's Medium (DMEM) supplemented with 10% fetal bovine serum (FBS), 2 mM L-glutamine and 1% penicillin-streptomycin (Gibco-BRL). The LMH (chicken) cells were purchased from ATCC and cultured in flasks pre-coated with 0.1% gelatin in DMEM supplemented with 10% FBS, 2 mM L-glutamine and 1% penicillin-streptomycin. Transient transfections were performed with TransIT-LT1 (Mirus), calcium phosphate (Clontech) or Lipofectamine 2000 (Invitrogen) according to the manufacturers' instructions.

### Plasmids

Mammalian expression constructs for untagged NS1 under the control of the chicken β-actin promoter (pCAGGS vector [45]) have been described previously for A/Puerto Rico/8/34 (PR8) and R38A/K41A mutant NS1 [41]), A/Swine/Texas/4199-2/98 (Sw/Tx/98), A/Puerto Rico/8/34 E96A/E97A mutant, and A/Hong Kong/156/1997 [38], and GST-PR8 and A/California/04/09 NS1 (Cal04) [40]. Human TRIM25-V5, human RIG-I and human GST-RIG-I 2CARD were described in [38]. Mouse TRIM25 was cloned from murine LA-4 cells by Superscript III one-step RT-PCR (Invitrogen) with forward primer (5′GAATTCACCATGGGCAAACCGATTCCGAACCCGCTGCTGGGCCTGGATAGCACCGCGGAGCTGAATCCTCTGGCC-3′) and reverse primer (5′CCTCTCTATCTGCTCCAAATAGGGATCC-3′). The forward primer includes an EcoRI site and V5 sequence, and the reverse primer includes a BamHI site. V5-tagged mouse TRIM25 cDNA was then subcloned into the EcoRI/BglII sites of the mammalian expression vector pCAGGS. Chicken TRIM25 was cloned from primary chicken embryo fibroblasts by Superscript III one-step RT-PCR with forward primer (5′- GAATTCACCATGGGCAAACCGATTCCGAACCCGCTGCTGGGCCTGGATAGCACCGCGACGTTGACCAAAGCTCAGTC-3′) and reverse primer (5′- GGCACTGTTCTCTCTCTCTGGTAACTCGAG-3′). The forward primer includes an EcoRI site and V5 sequence, and the reverse primer includes a XhoI site. V5-tagged chicken TRIM25 cDNA was then subcloned into the same sites of pCAGGS. For the generation of V5-tagged human/mouse TRIM25 chimera, the coiled-coil region of TRIM25 (aa191–379) was amplified using human TRIM25-V5 plasmid as template, and then ligated into the BstBI sites of the mouse TRIM25 sequence in the pCAGGS vector. The Myc/DDK-tagged pCMV6 mouse Riplet was purchased from Origene (Rockville, MD). The reporter plasmid carrying the firefly luciferase (FF-Luc) gene under the control of the IFN-β promoter (p125Luc) was kindly provided by Takashi Fujita (Kyoto University, Japan) [46]. The reporter plasmid carrying the *Renilla* luciferase gene (REN-Luc/pRL-TK) was purchased from Promega, WI. All sequences were verified by sequencing.

### Viruses

Sendai virus (SeV; Cantell strain) was obtained from Charles River Laboratories and propagated in 10-day old embryonated chicken eggs. The A/Puerto Rico/8/1934 virus was propagated in 8-day old, specific pathogen-free embryonated eggs (Charles River Laboratories; North Franklin, CT). Influenza virus titers were determined by plaque assay using MDCK cells.

Rescue plasmids encoding the NS segments of A/Swine/Texas/4199-2/98 and A/Hong Kong/156/1997, A/Texas/36/1991 and A/Panama/2007/1999 were generated by cloning the cDNA into the ambisense rescue plasmid pDZ [47]. The rescue plasmid encoding A/California/4/2009 NS was described previously [48]. Isogenic influenza A viruses (A/Puerto Rico/8/1934) harboring the NS segment of A/Swine/Texas/4199-2/98 or A/Hong Kong/156/1997 were rescued by reverse genetics as described elsewhere [38], [49]. The A/Puerto Rico/8/1934 viruses expressing the NS1 R38A/K41A or NS1 E96A/E97A mutant were described previously [38]. All viruses were amplified in 8-day old, specific pathogen-free embryonated hen's eggs (Charles River Laboratories; North Franklin, CT).

### Interferon Bioassay

Interferon bioassays were conducted as described previously [38], [50]. Immortalized mouse embryonic fibroblasts (MEF), seeded in 12-well plates, were infected with the indicated isogenic influenza viruses at a multiplicity of infection (MOI) of 2. At 12 h postinfection, supernatants were harvested and 2-fold dilutions of the influenza virus supernatants prepared. Diluted supernatants were then applied to bioassay using immortalized mouse fibroblast (L929) cells; At 20 h post-treatment, L929 cells were infected with VSV-GFP at an MOI of 2. GFP fluorescence was determined by fluorescence plate reader. Fluorescence values are reported on the y-axis as relative fluorescence units.

### GST Pull-Down Assay, Immunoprecipitation, and Immunoblot Analysis

HEK293T, Hepa1.6 and MEF cells were lysed in NP40 buffer (50 mM HEPES, pH 7.4, 150 mM NaCl, 1% [v/v] NP40 and protease inhibitor cocktail [Roche]) or RIPA buffer (50 mM TRIS-HCl pH 8.0, 150 mM NaCl, 1% [v/v] NP40, 0.5% sodium deoxycholate, 0.1% SDS, and protease inhibitor cocktail [Roche]). GST pull-down and immunoprecipitations were performed as described previously [25]. For immunoblotting, proteins were resolved by SDS-polyacrylamide gel electrophoresis (SDS-PAGE) and transferred onto a PVDF membrane (Immobilon-P Millipore or BioRad Laboratories). The following primary antibodies were used: anti-V5 (1∶2,000) (Invitrogen), anti-Flag (1∶5,000) (Sigma), anti-HA (1∶5,000) (Sigma), anti-GST (1∶2,000) (Sigma), anti-TRIM25 (monoclonal; 1∶2,000) (BD Biosciences), anti-TRIM25 (polyclonal; 1∶1,000) (Santa Cruz Biotechnology), anti-RIG-I (1∶1,000) (Alme-1, Alexis), anti-NS1 (1∶2000) [38], anti-β-actin (Abcam; Cambridge, MA). Immunoblots were developed with the following secondary antibodies: ECL anti-rabbit IgG horseradish peroxidase conjugated whole antibody from donkey, and ECL anti-mouse IgG horseradish peroxidase conjugated whole antibody from sheep (GE Healthcare; Buckinghamshire, England). The proteins were visualized by an enhanced chemiluminescence reagent (Pierce).

### Confocal Microscopy

HeLa cells were seeded into Lab-Tek II 8-well chamber slides (CC2 Glass slide, Nunc; Rochester, NY). After 12–16 h, plasmids harboring V5-tagged TRIM25, Myc-mRiplet, and/or NS1 plasmids were transfected with Lipofectamine 2000 (Invitrogen) at a ratio 1∶1. Six hours posttransfection the medium was changed. 24 h later, cells were washed with PBS, fixed with methanol-acetone (1∶1), permeabilized with 0.5% NP-40 (v/v) in PBS, and blocked with 0.5% BSA 0.2% fish gelatin in PBS. Cells were stained with anti-V5 or anti-Myc and anti-NS1 antibodies as well as DAPI. Secondary antibodies conjugated to Alexa-fluor 488 and Alexa-fluor 555 (Invitrogen) were used to visualize the proteins. Images were taken on a Leica SP5 DM confocal microscope (Leica Microsystems) at a magnification of 63×. Confocal laser scanning microscopy was performed at the MSSM-Microscopy Shared Resource Facility.

### IFN-β Luciferase Reporter Assay

Hepa1.6 cells, WT MEFs and *TRIM25 −/−* MEFs were transfected in 24-well plates (Falcon, Becton Dickinson, NJ) with 50 ng of IFN-β reporter plasmid, 20 ng of Renilla luciferase and 10 ng NS1 plasmids using Lipofectamine 2000 at a ratio 1∶2. 24 h later, cells were lysed and dual-luciferase assay was performed according to the manufacturer's instructions (Promega, Madison, WI, USA). Luciferase values were normalized to Renilla values, and the fold induction was calculated as the ratio of SeV-stimulated samples, or samples transfected with inducing plasmid versus samples transfected with empty plasmid (no stimulation).

### Direct Protein Interaction Assay

Recombinant human TRIM25 (described in [25]) and mouse TRIM25 purified from XL1blue bacteria as amino-terminal Maltose-Binding Protein (MBP) and carboxy-terminal Flag fusion proteins (MBP-h/mTRIM25-Flag) were incubated with bacteria-produced recombinant GST or GST-NS1 (PR8) in 50 mM Tris HCl, pH 7.4, 150 mM NaCl, and 0.1% NP40. TRIM25-NS1 protein complexes were then precipitated with Amylose agarose resin (New England Biolabs), and the binding reaction mix was incubated for 4 h at 4°C. Precipitated protein complexes were resolved by SDS-PAGE and visualized by Coomassie staining.

### siRNA-mediated Knockdown

Transient knockdown of endogenous Riplet in mouse Hepa 1.6 and MEF cells, seeded in 6-well plates, was achieved by transfection of siGenome SMARTpool siRNA specific for mouse Riplet (Dharmacon) with Lipofectamine and Plus reagent (Invitrogen) according to the manufacturer's instructions. A final concentration of 600 nM of mouse Riplet SMARTpool siRNA, or 600 nM of siGenome non-targeting siRNA (Dharmacon) was used per well. Riplet knockdown efficiency was determined by RT-PCR using specific primers.

Knockdown of endogenous human TRIM25 and Riplet in A549 cells was performed in 24-well plates by transfection with Lipofectamine RNAiMAX (Invitrogen). 10 picomols of siGenome SMARTpool siRNA specific for human TRIM25 or Riplet, or non-targeting control siRNA (Dharmacon) was used. 40 h later, cells were infected with the different influenza viruses at an MOI of 0.1 for 36 h. Cells were then harvested for qPCR analysis.

Knockdown of endogenous TRIM25 in chicken LMH cells (hepatocellular carcinoma cell line), seeded in 24-well plates, was achieved by transfection of a siRNA specific for chTRIM25 5′-GCGAGAUUUGCUGAGAGCUGAGUUU-3′ (Invitrogen). As control, LMH cells were transfected with a non-targeting control sequence 5′-AAGGACGCUGAGGCCUAAUCCUGUU-3′ (Invitrogen).

### Quantitative PCR

Total RNA was isolated using RNeasy kit (Qiagen) and subjected to DNAse digestion with Turbo DNase (Ambion). Reverse transcription was performed using the high capacity cDNA reverse transcription kit (Applied Biosystems). Real-time RT-PCR was performed in 384-well plates in triplicate using gene-specific primers and Lightcycler480 SYBR green I master mix (Roche) in a Roche LightCycler 480. Threshold cycles were calculated using the 2^nd^ derivative max value of every amplification curve. Relative mRNA abundances were calculated using the ΔΔCt method [51] using 18S rRNA as a reference and plotted as fold change compared to mock-control samples.

### Statistical Analysis

Statistical analysis was performed using Prism (Version 5.0, GraphPad Software, San Diego California USA). Student's paired t-test, or in defined cases Two-way ANOVA with Bonferroni post-test or Fisher's exact test were used. *p<0.05; **p<0.01; ***p<0.001.

## Supporting Information

Figure S1 **Amino acid sequence alignment of TRIM25 and NS1 proteins used in our study.** (**A**) Protein sequence alignment of the CCD of human (aa 211–353), mouse (aa 210–351), and chicken (214–355) TRIM25 proteins. The amino acid sequence of the TRIM25 CCDs was aligned using the Jalview software [52]. Amino acids in dark blue are conserved in the three sequences. Amino acids in light blue are identical in 2 out of 3 sequences. The predicted helical secondary structure, quality and consensus sequence are depicted under the alignments. (**B**) Protein sequence alignment of NS1 from human (A/California/04/09 [Cal04]), avian (A/Hong Kong/156/1997 [HK156]), swine (A/Swine/Texas/4199-2/98 [SwTx98]) and mouse-adapted (A/Puerto Rico/8/34 [PR8]) viruses. The amino acid sequence of cloned NS1 proteins was aligned using ClustalW2 (http://www.ebi.ac.uk/Tools/clustalw2/). Asterisks (*) indicate positions which have a single, fully conserved residue. Colons (:) indicate conservation between groups of strongly similar properties, and periods (.) indicate conservation between groups of weakly similar properties. The residues important for RNA binding and TRIM25 interaction are highlighted in blue and red, respectively [38]. Positions highlighted in grey indicate non-conserved amino acids.
(TIF)

Figure S2 **Lys172 in the 2CARD of human RIG-I is not conserved in mouse RIG-I.** Protein sequence alignment of mouse and human RIG-I 2CARD. Alignment was performed using ClustalW2 (http://www.ebi.ac.uk/Tools/clustalw2/). Asterisks (*) indicate positions which have a single, fully conserved residue. Colons (:) indicate conservation between groups of strongly similar properties, and periods (.) indicate conservation between groups of weakly similar properties. Positions highlighted in grey indicate non-conserved amino acids.
(TIF)

Figure S3 **Lys849 and Lys851 of human RIG-I are conserved in mouse RIG-I.** Protein sequence alignment of the C-terminal domain (CTD) of mouse and human RIG-I. Alignment was performed using ClustalW2 (http://www.ebi.ac.uk/Tools/clustalw2/). Positions highlighted in grey indicate non-conserved amino acids.
(TIF)

Figure S4 **(A) Endogenous RIG-I ubiquitination is markedly decreased upon influenza infection in WT and** ***TRIM25 −/−*** **MEFs.** WT and *TRIM25 −/−* MEFs were either mock-treated or infected with PR8 (MOI of 2) for 18 h. WCLs were subjected to IP with anti-RIG-I antibody, followed by IB with anti-Ub or anti-RIG-I antibody. **(B) Interaction of NS1 with mouse Riplet.** WCLs of mouse Hepa1.6 cells transfected with empty vector or Myc-tagged mouse Riplet (Myc-mRiplet) together with NS1-PR8, NS1-Cal04 or NS1-HK156 were subjected to IP with anti-Myc antibody, followed by IB with anti-NS1 or anti-Myc. **(C) The NS1 E96A/E97A mutant but not the R38A/K41A mutant interacts with mouse Riplet.** At 30 h posttransfection with empty vector or Myc-tagged mouse Riplet (Myc-mRiplet) together with NS1-PR8 R38A/K41A or E96A/E97A mutant, HEK293T WCLs were subjected to IP with anti-Myc antibody, followed by IB with anti-NS1 antibody.
(TIF)
