# Supplementary figures and images for: Species-Specific Inhibition of RIG-I Ubiquitination and IFN Induction by the Influenza A Virus NS1 Protein
Source: PLoS Pathog. 2012 Nov 29;8(11):e1003059. doi: 10.1371/journal.ppat.1003059 (PMC3510253; doi:10.1371/journal.ppat.1003059)

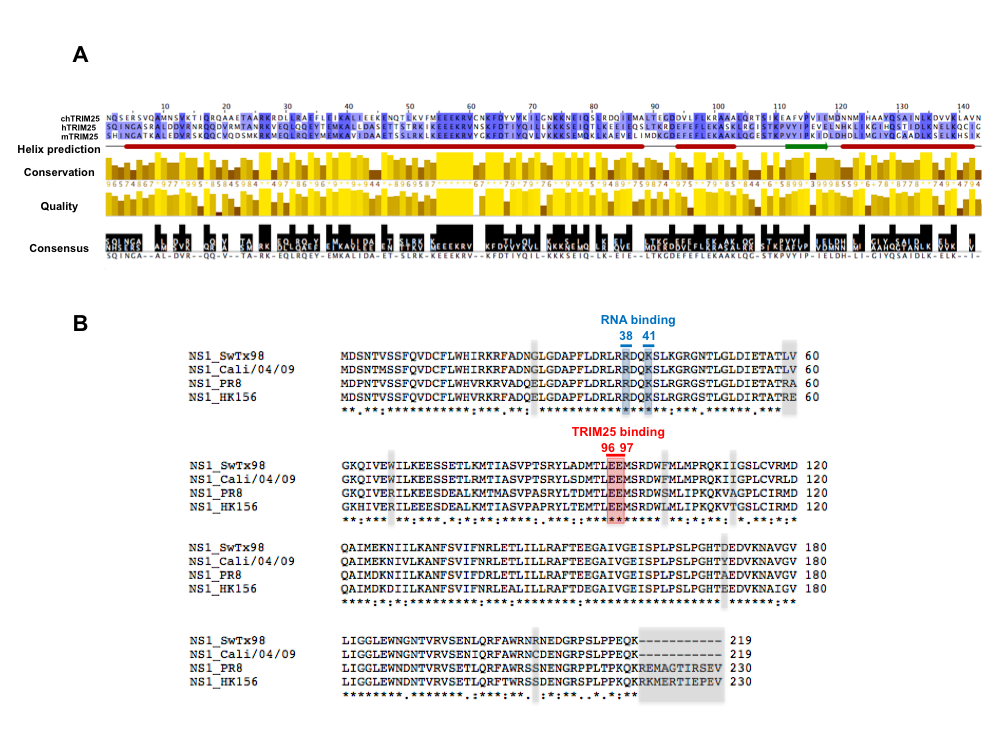

Supplement: Figure S1 — Amino acid sequence alignment of TRIM25 and NS1 proteins used in our study. (A) Protein sequence alignment of the CCD of human (aa 211–353), mouse (aa 210–351), and chicken (214–355) TRIM25 proteins. The amino acid sequence of the TRIM25 CCDs was aligned using the Jalview software [52]. Amino acids in dark blue are conserved in the three sequences. Amino acids in light blue are identical in 2 out of 3 sequences. The predicted helical secondary structure, quality and consensus sequence are depicted under the alignments. (B) Protein sequence alignment of NS1 from human (A/California/04/09 [Cal04]), avian (A/Hong Kong/156/1997 [HK156]), swine (A/Swine/Texas/4199-2/98 [SwTx98]) and mouse-adapted (A/Puerto Rico/8/34 [PR8]) viruses. The amino acid sequence of cloned NS1 proteins was aligned using ClustalW2 (http://www.ebi.ac.uk/Tools/clustalw2/). Asterisks (*) indicate positions which have a single, fully conserved residue. Colons (:) indicate conservation between groups of strongly similar properties, and periods (.) indicate conservation between groups of weakly similar properties. The residues important for RNA binding and TRIM25 interaction are highlighted in blue and red, respectively [38]. Positions highlighted in grey indicate non-conserved amino acids. (TIF) [file ppat.1003059.s001.tif]

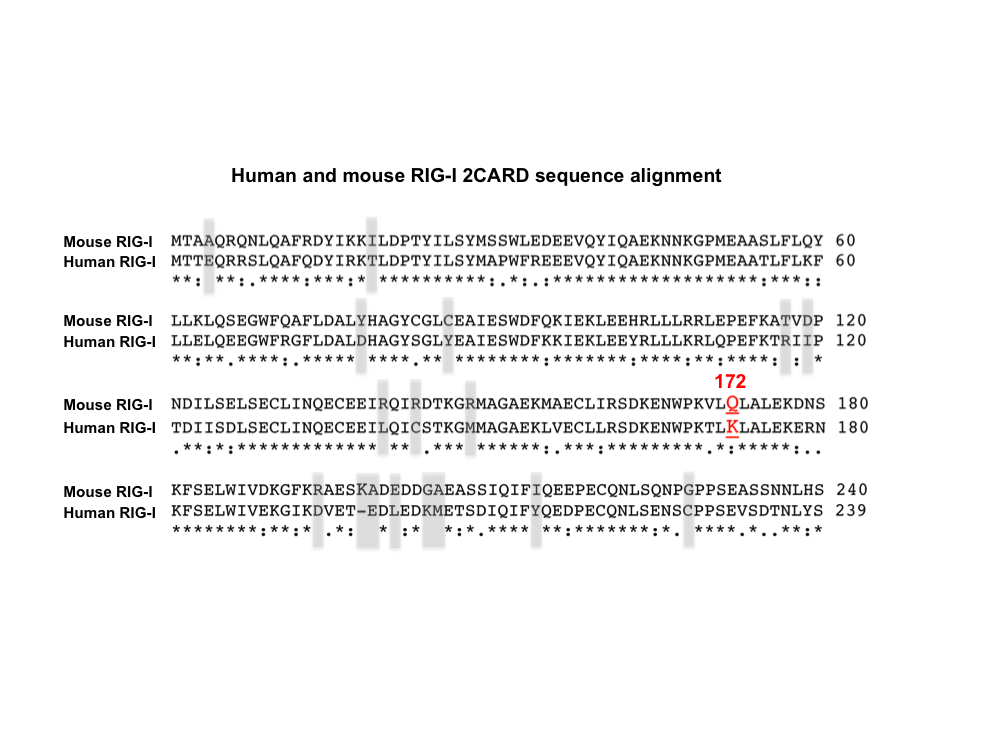

Supplement: Figure S2 — Lys172 in the 2CARD of human RIG-I is not conserved in mouse RIG-I. Protein sequence alignment of mouse and human RIG-I 2CARD. Alignment was performed using ClustalW2 (http://www.ebi.ac.uk/Tools/clustalw2/). Asterisks (*) indicate positions which have a single, fully conserved residue. Colons (:) indicate conservation between groups of strongly similar properties, and periods (.) indicate conservation between groups of weakly similar properties. Positions highlighted in grey indicate non-conserved amino acids. (TIF) [file ppat.1003059.s002.tif]

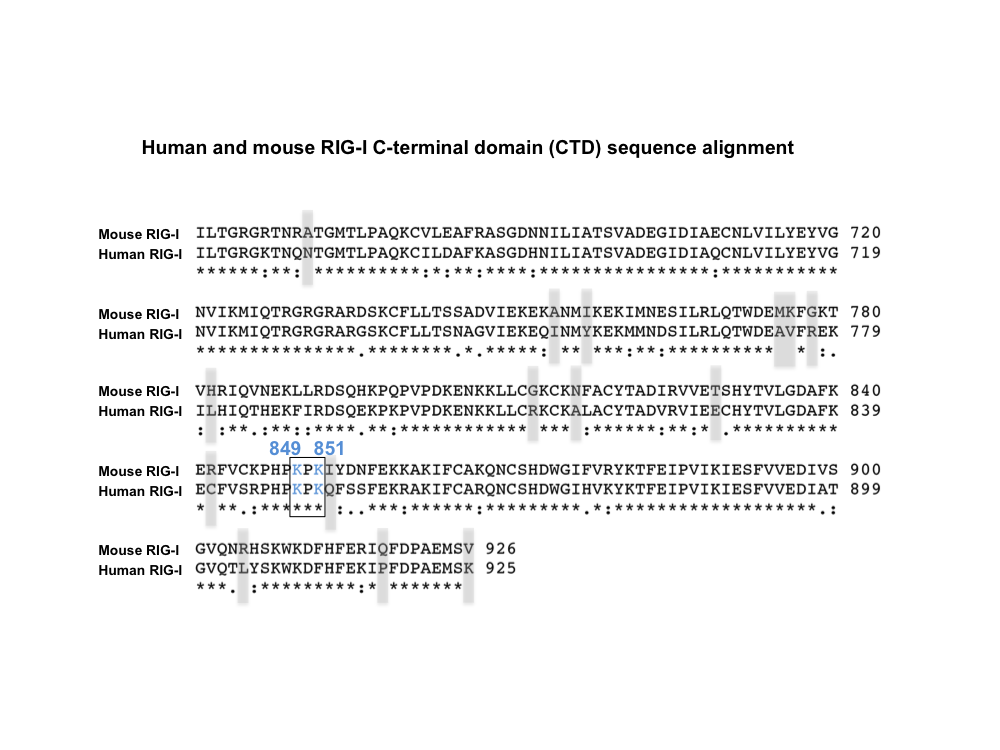

Supplement: Figure S3 — Lys849 and Lys851 of human RIG-I are conserved in mouse RIG-I. Protein sequence alignment of the C-terminal domain (CTD) of mouse and human RIG-I. Alignment was performed using ClustalW2 (http://www.ebi.ac.uk/Tools/clustalw2/). Positions highlighted in grey indicate non-conserved amino acids. (TIF) [file ppat.1003059.s003.tif]
